# Editorial: Assessing and evaluating the psychosocial impact of the COVID-19 pandemic on anxiety and stress: perspectives from East Asia

**DOI:** 10.3389/fpsyt.2023.1353718

**Published:** 2024-01-05

**Authors:** Qiao Zhou, Jingying Wang, Wenjie Duan, Baojuan Ye

**Affiliations:** ^1^Social and Public Administration School, East China University of Science and Technology, Shanghai, China; ^2^School of Psychology, Jiangxi Normal University, Nanchang, China

**Keywords:** COVID-19, East Asia, psychosocial impact, anxiety and stress, pandemic

## Introduction

Large-scale epidemics like COVID-19 often trigger panic and anxiety in the public (1). In fact, a number of studies have shown that the epidemic has triggered a massive mental health crisis across the globe (2). In the case of East Asia, people have experienced prolonged quarantine and lockdown measures, which had a profound impact on their lives (3). It has been proved that the extended duration of quarantine and lockdown during the COVID-19 outbreak period led to increased feelings of anxiety and stress among the public (4). For example, an online survey conducted in China found that during the peak of the epidemic, about 35% of respondents reported that they felt moderate to extreme psychological stress, and about 20% reported an increase in their anxiety compared to the norm (5).

COVID-19 profoundly impacted mental health, social interactions, and lifestyle (6). For example, during periods of quarantine, there can be a negative impact on the quality of a person's life, which can affect psychological burdens (7). Even cause serious mental health problems, such as posttraumatic stress symptoms (8). Still, it also caused social exclusion negatively related to control over COVID-19 threat and quality of life (9). Meanwhile, the virus caused stigmatization of potentially infected individuals and harm their mental health and social relationships, especially in Hubei (10).

The East Asian region has accumulated considerable experience in dealing with infectious diseases, such as the 2003 SARS epidemic, resulting in significant achievements in public health policy and institution-building in these countries and regions from a historical perspective (11). The Japanese government swiftly implemented a series of countermeasures against COVID-19, including travel restrictions, activity limitations, and temporary school closures (12). Although strict epidemic prevention policies and control measures yielded significant results, their consequences are increasingly evident. Prolonged quarantine, travel restrictions, and social distancing may heighten the risk of psychological distress, including anxiety and depression (13).

Coping styles in East Asia should be adapted to cultural contexts and population characteristics. Western cultures rely on sharing emotions and seeking support to cope with stress (14). In collectivist cultures in East Asia, people may deal with emotions more through internalization, deep reflection and dealing with emotions alone, avoiding causing problems for others in the process (15). Specifically, introspective and self-adjustment approaches may be effective in promoting mental health in East Asia. In China, utilizing family support networks and community resources is suggested as an effective way to cope with epidemic stress (16). Coping COVID-19 can be improved by boosting family psychological support and utilizing community resources like hotlines. Stress and anxiety management in East Asia should suit its cultural and population traits.

## Structure and contribution of the Research Topic

The manuscripts in this Research Topic are summarized in Table 1, and we we've made some comments about these manuscripts in this editorial.

**Table 1:** Summary of contributions to the Research Topic.

**No**	**References**	**Title**	**Purpose**	**Views**
1	Sun et al.	*COVID-19 Pandemic-related Depression and Anxiety under Lockdown: The Chain Mediating Effect of Self-Efficacy and Perceived Stress*	The objective of this study was to explore the prevalence and associated factors of depression and anxiety in isolated or quarantined populations under lockdown.	819
2	Wang T. et al.	*The Relationship Between Intolerance of Uncertainty, Coping Style, Resilience, and Anxiety During the COVID-19 Relapse in Freshmen: A Moderated Mediation Model*	The innovation of this study is the first to explore the mechanism of coping style and resilience as people's psychological protective factors between uncertainty and anxiety caused by the COVID-19 pandemic.	1,577
3	Geng et al.	*Emotional Distress and Burnout at A Fever Clinic in China: Comparison Between Different Periods of Covid-19*	The aim of this study was to examine the psychological symptoms and occupational burnout of FHWs in a fever clinic during different periods of the pandemic.	526
4	Yin et al.	*Posttraumatic Stress Disorder Symptoms Among Healthcare Workers During the Omicron Era*	The objective of this study was to clarify the factors that influenced health workers' posttraumatic stress disorder (PTSD) symptoms.	676
5	Tan et al.	*Anxiety Among Hospitalized Covid-19 Patients: A Case–Control Study from a Tertiary Teaching Hospital in Malaysia*	The aim of this study was to estimate the prevalence of and risk factors of anxiety in COVID-19 patients compared to controls in a local tertiary teaching hospital in Malaysia.	779
6	He et al.	*Comparison of Peripheral Blood T, B, and NK Lymphocytes Between Frontline Medical Workers for Treating Patients of Covid-19 and Normal Outpatient and Emergency Medical Workers in China*	The aim of this study was to compare the differences in mental health and immune function between 158 frontline medical workers and 24 controls from medical staffs of the outpatient and emergency departments.	693
7	Tian et al.	*Translation, Adaptation, and Initial Evaluation of a Guided Self-Help Intervention to Reduce Psychological Distress Among Nurses During Covid-19 in China*	The objective of this study was to translate and adapt the SH+ guideline into the Chinese version and to test its feasibility in reducing psychological distress among nurses during COVID-19.	560
8	Ding et al.	*A Multifactorial Framework of Psychobehavioral Determinants of Coping Behaviors: An Online Survey at the Early Stage of the Covid-19 Pandemic*	The objective of this study was to try to identify the major coping-behavior and risk-perception factors. And then examined important demographic, risk-perception, and psychobehavioral factors that contributed to coping behavior.	541
9	Wang Y. et al.	*A Decline in Perceived Social Status Leads to Post-Traumatic Stress Disorder Symptoms in Adults Half a Year After the Outbreak of the Covid-19 Pandemic: Consideration of the Mediation Effect of Perceived Vulnerability to Disease*	The aim of this study was to examine the impact of perceived social status decline on the prevalence of PTSD symptoms and check the mediating effect of perceived vulnerability to disease (PVD) during the period of psychological adjustment.	704
10	Guo Z. et al.	*Family Function and Anxiety Among Junior School Students During the Covid-19 Pandemic: A Moderated Mediation Model* *Perceived Covid-19 Stress and Online Aggression Among Chinese First-Year College Students: A Moderated Mediation Model*	The purpose of this study was to explore the mediating and moderating mechanisms underlying this relationship among junior school student during the COVID-19 pandemic.	542
11	Guo L. et al.	*Perceived COVID-19 Stress and Online Aggression Among Chinese First-Year College Students: A Moderated Mediation Model*	The aim of this study was to examine a moderated mediation model with anxiety as a mediator and perceived anonymity as a moderator.	642
12	Wan et al.	*Psychological Resilience Matters in the Relationship Between the Decline in Economic Status and Adults' Depression Half a Year After the Outbreak of the COVID-19 Pandemic*	This article studied the prevalence of depression among the population of Hubei Province since the pandemic is of great significance.	611

### Factors associated with anxiety and stress and the relationship between different variables during the COVID-19 pandemic

The following three articles examine the associations between COVID-19, anxiety, and stress.

In the first article, Sun et al. examined depression and anxiety prevalence and factors in isolated or quarantined populations under lockdown. Results showed that higher education, healthcare worker infections, prolonged isolation, and high perceived stress levels were risk factors. The study also found an association between perceived social support and depression/anxiety, mediated by perceived stress and self-efficacy. The study recommends psychological strategies promoting social support and self-efficacy to alleviate depression and anxiety in isolated or quarantined populations.

The second article investigated the role of coping styles and resilience in the face of uncertainty and anxiety caused by COVID-19. Wang T. et al. explored the relationship between uncertainty tolerance, anxiety, and coping styles. Results found that the tested students had higher anxiety scores than the Chinese standard, and uncertainty tolerance was positively correlated with anxiety. Positive coping styles had a negative impact on anxiety, while negative coping styles had a positive impact. Resilience moderated the effect of negative coping styles on anxiety. The study concluded that high uncertainty tolerance reduced psychological burdens during the pandemic. Healthcare workers can utilize coping styles and resilience knowledge to counsel and assist students with physical discomfort and psychosomatic disorders.

Guo L. et al. explored the link between COVID-19 stress, anxiety, and cyber-aggressive behaviors. They utilized a moderated mediation model to investigate the underlying factors, with anxiety as the mediator and perceived anonymity as the moderator. A survey of 3,069 first-year Chinese university students assessed COVID-19 stress, anxiety, cyber-aggressive behavior, and perceived anonymity. Findings revealed a positive relationship between perceived stress and online aggressive behavior, moderated by anxiety. Additionally, perceived anonymity further moderated the relationship between stress/anxiety and online aggressive behavior. The study suggests implementing psychological strategies to alleviate anxiety and perceived anonymity, and to address online aggression during COVID-19.

The above three articles focused on the link between COVID-19, anxiety, and stress in non-healthcare groups. The following four articles focused on changes in the psychophysiological status of healthcare workers and patients, along with influencing factors and relationships.

Geng et al. analyzed psychological symptoms and burnout among frontline healthcare workers in fever clinics during various COVID-19 pandemics. The study included 162 participants surveyed during pandemic and non-pandemic periods. Results indicated prevalent anxiety, depressive symptoms, and burnout among healthcare workers. Although depression decreased as the pandemic severity lessened, anxiety and burnout remained high. Self-efficacy was identified as a crucial factor in protecting frontline healthcare workers from burnout. The study recommended the development of institutional support and intervention programs for these workers.

The second article aimed to assess the prevalence and risk factors of anxiety disorders in COVID-19 patients hospitalized in a Malaysian teaching hospital. Tan et al. compared adult COVID-19 patients with a hospitalized control group and found significantly higher prevalence of anxiety disorders among the COVID-19 patients. The severity of GAD-7 was also notably higher in the COVID-19 group. COVID-19 diagnosis and neurologic symptoms were identified as significant predictors of patient anxiety. The study recommended early mental health attention and psychiatric referral for COVID-19 patients.

In the next article, He et al. compared CD3, CD4, CD8, CD19, and CD56 lymphocytes in 158 frontline medical staff and 24 outpatient medical staff to assess immune function changes in those treating COVID-19 patients. The study found significantly lower absolute values and percentages of CD19+ B-cells in frontline medical staff, especially in females and those over 40. Additionally, lower absolute CD4+ T cell values were observed in medical staff under 40, while those over 40 showed lower CD8+ T cell percentages and higher CD56+ NK cell percentages. The study underscores the importance of prioritizing mental health and immune function in frontline medical staff, along with providing suitable psychological support and care measures.

In the final article in this section explored factors influencing posttraumatic stress disorder (PTSD) symptoms among healthcare workers in the COVID-19 pandemic. Using data from 443 workers in Shandong Mental Health Centers, the research found that 45.37% exhibited severe PTSD symptoms. High exposure to COVID-19 was directly linked to symptom severity, while euthymia and perceived social support were inversely correlated. Yin et al. also found that exposure to COVID-19 partially mediated PTSD symptoms through euthymia and was moderated by social support from friends, leaders, relatives, and colleagues. Enhancing euthymia and bolstering social support could alleviate PTSD symptoms in healthcare professionals during the COVID-19 crisis.

During the epidemic, people faced numerous social, demographic, and economic challenges that exacerbated anxiety and stress. The following three articles focus on social, demographic and economic factors related to anxiety and stress during the epidemic.

In the first article, based on cognitive-relational theory, Wang Y. et al. studied how perceived social status decline affects PTSD symptoms through perceived disease susceptibility (PVD) during mental adjustment. Findings reveal lower social status correlating with worsened PTSD, while PVD offered slight buffering. Emphasizing subjective social status in health outcomes, the study suggests improving community social support to boost mental health perceptions.

The second one, Guo Z. et al. investigated family function, anxiety, mediators, and moderators in 745 middle students during COVID-19. Homebound students reported lower family function, higher stress, and anxiety. Results showed: (1) Left-behind (LB) junior students had lower function, higher stress, and anxiety; (2) Family function negatively linked to anxiety with stress as mediator; (3) LB status moderates the connection between function, anxiety, and stress. These findings enhance anxiety prevention in middle schoolers during the pandemic.

In the final article in this section, Wan et al. examined the connection between economic status decline and adult depression in the 6 months post-COVID outbreak, focusing on the role of psychological resilience. Findings indicated that depression severity heightened as economic status declined, with each unit drop relating to an approximate 0.117 unit increase in depression severity. Also, psychological resilience was found to play a significant moderating role. The study emphasizes the impact of economic status on depression and proposes solutions to improve mental health during pandemics.

### Coping styles used to cope with anxiety and stress during the COVID-19 epidemic and their coping outcomes

This section discusses various coping strategies that can be developed and encouraged to enhance and maintain individuals' physical and mental health during an epidemic, along with the effects resulting from these coping styles.

The first article aimed to use the World Health Organization's Self-Help Plus (SH+) intervention, adapted for the Chinese context, to alleviate nurses' psychological distress during the COVID-19 pandemic. The study conducted in two Xi'an hospitals involved translation, adaptation, and evaluation through a pilot implementation involving 20 nurses. Results showed significant reductions in distress, improvements in psychological flexibility, wellbeing, and depressive symptoms. Despite adherence difficulties, Tian et al. found the Chinese SH+ version feasible for use in China and potentially helpful for nurses during COVID-19, but recommended exploring strategies to improve adherence.

The second article by Ding et al. aimed to identify factors impacting coping-behavior during a pandemic. Using hierarchical multiple regression analyses, it identified four coping-behaviors and three risk perception factors and their correlations. It revealed positive correlations between different coping behaviors and factors such as femininity, rituals, risk perception, leadership, etiquette, and wellbeing. This simplified model aids in understanding social dynamics during a pandemic and offers a theoretical framework for coping behaviors.

## Future research

The study focuses on the mental health impact of COVID-19 on East Asia's general population and healthcare workers' resilience. It delves into the psychological shifts, influential factors, and adaptability modes in response to the pandemic. Findings indicate that education, job status, isolation duration, and perceived stress can affect mental health. Enhancing social support, self-efficacy, and reducing stress improves mental health. Coping strategies include self-help interventions and resilience training, requiring appropriate translation for East Asian groups to increase intervention efficiency.

Future studies should explore the epidemic's mental health impacts and devise more effective coping strategies for the public and healthcare professionals. They could utilize longitudinal studies to gauge when interventions are most effective. Research should also focus on unique groups like the youth, elderly, and disabled, who might require specialized interventions. Additionally, assessing the measures' long-term efficacy and sustainability in improving mental health is crucial.

## Author contributions

QZ: Writing – original draft. JW: Writing – original draft. WD: Writing – review & editing. BY: Writing – review & editing.

## References

[1] Bao Y Sun Y Meng S Shi J Lu L. 2019-ncov epidemic: address mental health care to empower society. Lancet. (2020) 395:e37–8. 10.1016/S0140-6736(20)30309-3 32043982 PMC7133594

[2] Rajkumar RP. COVID-19 and mental health: a review of the existing literature. Asian J Psychiatr. (2020) 52:102066. 10.1016/j.ajp.2020.102066 32302935 PMC7151415

[3] Cao W Fang Z Hou G Han M Xu X Dong J. The psychological impact of the COVID-19 epidemic on college students in china. Psychiatry Res. (2020) 287:112934. 10.1016/j.psychres.2020.112934 32229390 PMC7102633

[4] Wang C Pan R Wan X Tan Y Xu L Ho CS. Immediate psychological responses and associated factors during the initial stage of the 2019 coronavirus disease (COVID-19) epidemic among the general population in china. Int J Environ Res Public Health. (2020) 17:1729. 10.3390/ijerph17051729 32155789 PMC7084952

[5] Qiu J Shen B Zhao M Wang Z Xie B Xu Y. A nationwide survey of psychological distress among chinese people in the COVID-19 epidemic: implications and policy recommendations. General Psychiat. (2020) 32:e100213. 10.1136/gpsych-2020-100213 32215365 PMC7061893

[6] Li J Fong DYT Lok KYW Wong JYH Man Ho M Choi EPH. Global impacts of covid-19 on lifestyles and health and preparation preferences: an international survey of 30 countries. J Global Health. (2023) 13:06031. 10.7189/jogh.13.06031 37565394 PMC10416140

[7] Zhang Z Wang J Duan W. The impact of adolescents' character strengths on quality of life in stressful situations during COVID-19 in china: a moderated mediation approach. J Evid Based Soc Work. (2023) 20:881–95. 10.1080/26408066.2023.2231438 37395636

[8] Duan W Guan Q Jin Q. Latent profiles and influencing factors of posttraumatic stress symptoms among adults during the COVID-19 pandemic. Front Public Health. (2021) 9:620521. 10.3389/fpubh.2021.620521 34249828 PMC8264580

[9] Duan W Kong Y Chen Z Mu W. COVID-19-induced social exclusion and quality of life among chinese adolescents in the context of family education: the mediating role of perceived control. Educ Psychol. (2022) 43:531–44. 10.1080/01443410.2022.2099529

[10] Duan W Bu H Chen Z. COVID-19-related stigma profiles and risk factors among people who are at high risk of contagion. Soc Sci Med. (2020) 266:113425. 10.1016/j.socscimed.2020.113425 33059301 PMC7540249

[11] Bouey J. Strengthening china's public health response system: from sars to COVID-19. Am J Public Health. (2020) 110:939–40. 10.2105/AJPH.2020.305654 32213081 PMC7287547

[12] Shimizu K Negita M. Lessons learned from japan's response to the first wave of COVID-19: a content analysis. Healthcare. (2020) 8:426. 10.3390/healthcare8040426 33114264 PMC7711542

[13] Tull MT Edmonds KA Scamaldo KM Richmond JR Rose JP Gratz KL. Psychological outcomes associated with stay-at-home orders and the perceived impact of covid-19 on daily life. Psychiatry Res. (2020) 289:113098. 10.1016/j.psychres.2020.113098 32434092 PMC7252159

[14] Tamres LK Janicki D Helgeson VS. Sex differences in coping behavior: A meta-analytic review and an examination of relative coping. Person Soc Psychol Rev. (2016) 6:2–30. 10.1207/S15327957PSPR0601_1

[15] Kim HS Sherman DK Taylor SE. Culture and social support. Am Psychol. (2008) 63:518–26. 10.1037/0003-066X 18793039

[16] Yi Y Lagniton PNP Ye S Li E Xu R-H. COVID-19: what has been learned and to be learned about the novel coronavirus disease. Int J Biol Sci. (2020) 16:1753–66. 10.7150/ijbs.45134 32226295 PMC7098028

